# Enhanced physical performance and quality of life in cardiovascular disease patients across BMI groups through exercise-based cardiac rehabilitation

**DOI:** 10.3389/fcvm.2026.1875972

**Published:** 2026-07-09

**Authors:** Xinmeng Liu, Guangxin Liu, Zibo Wang, Jingxiang Zhao, Deyu Qin, Chaodong Pu, Qian Zhang, Ying Zhang, Mei Ma

**Affiliations:** 1 Department of Rehabilitation Medicine, Tianjin Chest Hospital, Tianjin, China; 2 Department of Medical Equipment, Tianjin Chest Hospital, Tianjin, China

**Keywords:** body mass index, cardiac rehabilitation, cardiovascular diseases, exercise, quality of life

## Abstract

**Background and aims:**

In the face of escalating cardiovascular disease (CVD) prevalence, the demand for evidence-backed cardiac rehabilitation has surged, making it a guideline-recommended secondary prevention and adjunctive management strategy for afflicted individuals.

**Methods:**

Our study meticulously investigated the effects of a structured cardiac rehabilitation (CR) program on 79 CVD patients. These participants engaged in a carefully designed regimen, including 12 in-center sessions held 2–3 times per week, with comprehensive assessments conducted both before and after the intervention. These assessments encompassed a cardiopulmonary exercise test (CPET) and the completion of four questionnaires targeting aspects of quality of life and mental health, specifically depression and anxiety levels.

**Results:**

Following the cardiac rehabilitation, oxygen uptake (VO_2_, 10.9 ± 2.9 to 13.3 ± 3.4 mL/min/kg, *p* < 0.01), ventilation (VE, 29.3 ± 7.5 to 35.6 ± 9.3 mL/min/kg, *p* < 0.01), workload (56.7 ± 25.4 to 70.0 ± 27.7 W, *p* < 0.01) at the anaerobic threshold (AT), and the oxygen uptake efficiency slope (OUES, 1,426.8 ± 346.3 to 1,547.2 ± 403.5, *p* < 0.01) significantly improved in CVD patients. These positive outcomes were consistent across study groups. Additionally, participants experienced significant enhancements in quality of life, coupled with reduced depression and anxiety levels post-rehabilitation.

**Conclusion:**

Collectively, the present study corroborates compelling evidence that exercise-driven cardiac rehabilitation confers meaningful improvements in physical performance and quality of life for patient cohorts irrespective of body mass index.

## 1 Introduction

Cardiovascular diseases (CVD) encompass a range of conditions, including ischemic heart disease, stroke, heart failure, peripheral artery disease, and other heart and vascular conditions. These diseases are the leading cause of global mortality and significantly impact quality of life (1, 2). In 2017, CVD were responsible for an estimated 17.8 million deaths worldwide, resulting in a loss of 330 million years of life and an additional 35.6 million years lived with disability (3, 4). In China, CVD account for a staggering 40% of all deaths, positioning the country with one of the highest prevalence rates of CVD (3, 5, 6).

CVD significantly affect the cardiorespiratory function and overall quality of life (QoL) for individuals. Patients with CVD experience increased healthcare needs and a reduction in independence (7). Moreover, aerobic capacity, a fundamental aspect of physical performance, naturally declines with age, typically diminishing by 8%–10% per decade of life (8, 9). This decline in aerobic capacity also hampers the ability to perform everyday activities, potentially leading to a cycle of sedentary behavior (10). Simultaneously, CVD often trigger negative emotions such as depression and anxiety, further affecting the overall quality of life (11, 12).

The American Heart Association (AHA) and American College of Cardiology (ACC) consider cardiac rehabilitation (CR) as a Class I indication for various conditions such as acute coronary syndrome (ACS), percutaneous coronary intervention (PCI), coronary artery bypass grafting (CABG), valve surgery, and chronic stable heart failure with reduced ejection fraction (13, 14). CR is a comprehensive and multidimensional treatment program that typically includes medical evaluation, exercise training, modification of cardiac risk factors, and education. It aims to promote lifelong health and wellness in individuals with CVD (15, 16). One of the key objectives of CR is to assist patients in the transition from the hospital, providing supervised physical activity to minimize deconditioning associated with illness and hospitalization, while also offering valuable support and education.

Body Mass Index (BMI) plays a crucial role in evaluating cardiovascular health and its impact on cardiac rehabilitation (17). A high BMI, indicative of obesity, is a well-established risk factor for the development and progression of various cardiovascular conditions, such as hypertension, dyslipidemia, and type 2 diabetes (18, 19). Concerning its influence on cardiac rehabilitation, individuals with elevated BMIs often encounter distinctive challenges throughout the rehabilitation process. These challenges manifest as limitations in physical endurance and a heightened risk of joint problems, thereby complicating the execution of certain exercises (20, 21). However, it is noteworthy that the current body of research exploring the effectiveness of cardiac rehabilitation for patients with varying BMIs is limited. More studies are required to comprehensively understand how rehabilitation interventions can be optimized for individuals with different BMI levels.

The aim of this study was to assess the impact of exercise-based cardiac rehabilitation on patients with varying BMI levels and to investigate its potential to enhance cardiorespiratory fitness, thereby improving physical performance and overall quality of life in individuals with cardiovascular disease.

## 2 Materials and methods

### 2.1 Study design

**Figure 1:**
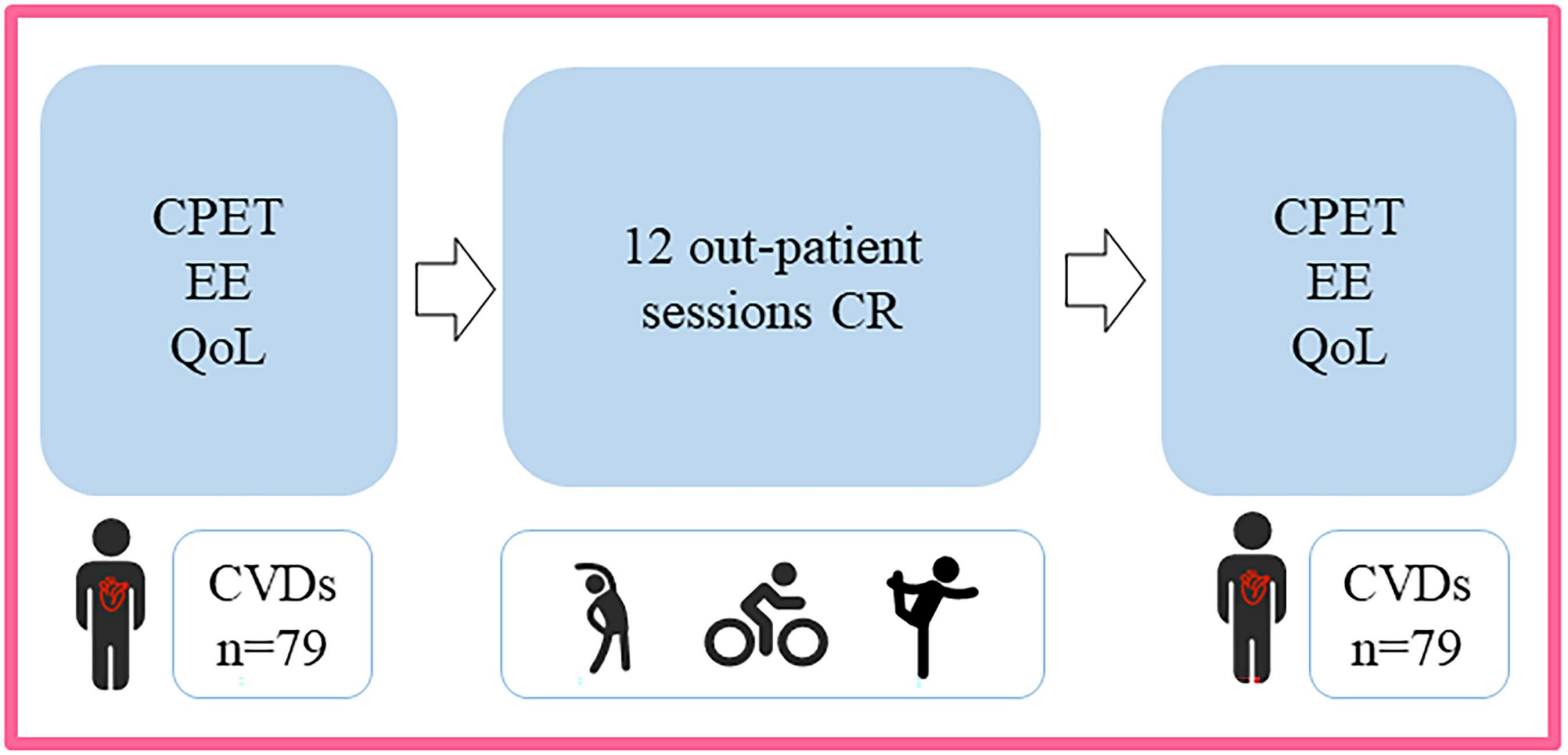
Flowchart of this study. CPET, cardiopulmonary exercise testing; EE, energy expenditure; QoL, quality of life; CR, cardiac rehabilitation.

This was a prospective, single-arm, pre-post interventional study conducted among patients with CVD at Tianjin Chest Hospital from December 2020 to July 2021.The study protocol received approval from the local ethics committee [IRB-SOP-016(F)-001-02, 9 August 2021]. We provided a detailed explanation of the study's purpose and methods to all staff, including cardiologists, physical therapists, and nurses. Prior to enrollment, all subjects signed informed consent forms. The entire trial consisted of twelve supervised outpatient sessions held 2 or 3 times a week, along with home exercise and two clinical assessments conducted before and after the CR interventions. Primary outcomes: VO_2_ at anaerobic threshold (AT) and oxygen uptake efficiency slope (OUES). Secondary outcomes: VE at AT, workload at AT, VE/VCO_2_ slope, energy expenditure (EE), SF-12 score. Exploratory outcomes: GAD-7, PHQ-9, and sleep quality (PSQI). Figure 1 presents a flowchart depicting the study's design.

### 2.2 Study subjects

Subjects were recruited from patients with CVD. Inclusion criteria: (1) age >18 years; (2) diagnosed with CVD (coronary heart disease, old myocardial infarction, arrhythmias, or heart valve disease); (3) no prior PCI or at least one-week post-PCI; (4) no prior CABG or at least one-month post-CABG. Exclusion criteria: abnormal blood pressure response, acute heart failure, unstable angina, acute myocardial infarction, congenital heart disease, or severe musculoskeletal diseases limiting exercise.

### 2.3 Outpatient rehabilitation protocol

The rehabilitation program was designed to address various aspects of patient well-being, including the control of cardiovascular risk factors, diet monitoring, therapeutic education sessions, and psychological support when needed. It aimed to provide comprehensive care to patients on their path to recovery.

**Intensity prescription:** Exercise intensity was prescribed at the heart rate and workload corresponding to the anaerobic threshold (AT) determined by the initial CPET. The target heart rate zone was set as AT heart rate ± 5 bpm. The initial exercise intensity in the first week was 40%. If the patient experienced no discomfort, the intensity was increased by 10%–15% weekly until the target heart rate was achieved.

**Monitoring:** Each session was continuously monitored via 12-lead telemetry (GE Healthcare, Chicago, IL, USA). Blood pressure was measured before, during (every 15 min), and after exercise.

**Home exercise adherence:** Home-based exercise consisted of 15 min of breathing training and warm-up, 30 min of brisk walking, and 15 min of stretching. We provided instructional videos for breathing training and warm-up exercises. Patients used their own smart bands to monitor heart rate, and the exercise intensity was kept consistent with that applied during outpatient rehabilitation sessions of the current week. The training was performed three times a week.

**Adverse events:** No major adverse events (e.g., myocardial infarction, cardiac arrest, or hospitalization) occurred during the 12 supervised sessions. Minor events (e.g., muscle soreness, mild dyspnea) were reported by 4 patients (5.0%) and did not require intervention.

### 2.4 Measurement

#### 2.4.1 Cardiopulmonary exercise test (CPET)

The cardiopulmonary exercise test was conducted under the supervision of a specialist cardiologist according to current guidelines for CVD patients. A continuous progressive exercise protocol was performed on a cycle ergometer (Oxycon Mobile, JAEGER-CareFusion, Hoechberg, Germany). Individualized ramp protocol was used for CPET. Oxygen VO_2_ and carbon dioxide (CO_2_) were registered by a breath-by-breath analysis. HR, VO_2_, metabolic equivalent of task (MET), respiratory exchange ratio (RER) and ventilation (VE) were collected at resting state and AT. ECG activity was continuously monitored by 12-lead ECG and was recorded throughout the test and during the 6-min passive recovery period. The power of the bicycle at AT in CPET was also collected to evaluate physical performance in participants. The anaerobic threshold (AT) was determined independently by two experienced cardiologists using the V-slope method, with concordance required. The maximal effort criterion was set as a respiratory exchange ratio (RER) > 1.05, which is a clinically accepted threshold for patients with cardiovascular disease to ensure safety while still indicating near-maximal effort, as conventional thresholds (>1.10–1.15) may not be attainable or safe in this population (22, 23).

#### 2.4.2 Energy expenditure

Energy expenditure was calculated using the Weir equation: EE (kcal/d) = [1.59 × VCO_2_ (mL/min) + 5.68 × VO_2_ (mL/min) + 2.17 × Nu] × 1.44, where Nu is a fixed value for urinary nitrogen excretion (set at 13 g/d based on a standard protein intake of 81 g/d for our population).

#### 2.4.3 Quality of life (QoL)

QoL was assessed using the self-report questionnaire SF-12, with scores ranging from 0 to 100 where higher scores represented better QoL. Additionally, level of anxiety assessed by Generalized Anxiety Disorder 7-item (GAD-7), level of depression assessed by Patient Health Questionnaire-9 (PHQ-9). GAD-7 and PHQ-9 were grade variables, which were respectively divided into 4 groups and 5 groups. Higher score presented higher severity of anxiety or depression. Sex, age and complications were registered in questionnaires and patient records. We assessed the patients' depressed mood using the Depression Measurement Scale, where higher scores indicate a greater propensity for depression. Similarly, we evaluated the patients' sleep quality using the Pittsburgh Sleep Quality Index, which examines seven aspects of sleep quality, including time to sleep, sleep duration, and sleep efficiency. In this case, higher scores on the scale are indicative of poorer sleep quality.

### 2.5 Statistical analysis

A priori sample size calculation was performed based on a paired t-test, using an expected mean difference in VO_2_ at AT of 2.0 mL/min/kg (SD = 3.0), with 80% power and a two-sided alpha of 0.05. This required a minimum of 44 patients to detect a significant change. We enrolled 79 patients to account for potential dropouts and to allow for subgroup analyses. Normality of continuous variables was assessed using the Shapiro–Wilk test. Paired t-tests were used for within-group comparisons of normally distributed data. For non-normally distributed data, the Wilcoxon signed-rank test was applied. For between-subgroup comparisons (high vs. low BMI), two-sample t-tests or Mann–Whitney *U* tests were used, as appropriate. There were no missing data for the primary outcomes; all 79 patients completed both assessments. For secondary questionnaires, missing responses (*n* = 3 for PHQ-9) were handled using multiple imputation. To correct for multiple comparisons across the 10 primary and secondary outcomes, a false discovery rate (FDR) of 0.05 was applied using the Benjamini-Hochberg procedure. All reported *p*-values are FDR-adjusted.

## 3 Results

### 3.1 Study group characteristics

In our study, all 79 enrolled patients were eligible for analysis. Table 1 provides an overview of the demographic characteristics and clinical profiles of the patients. The majority of patients (78.48%) were male, with a mean age of 53.53. Additionally, a significant percentage (74.68%) of participants were overweight (BMI > 24.0 kg/m^2^), and 25.31% were classified as obese (BMI ≥ 28.0 kg/m^2^). Among the participants, 65 (82.27%) had coronary heart disease, 36 (45.56%) had experienced myocardial infarction, 17 (21.51%) had arrhythmias, and 5 (6.32%) had heart valve disease. Furthermore, 39 patients had concomitant hypertension, while 12 patients had diabetes. Regarding surgical history, it was found that more than one-third of patients (40.51%) had undergone percutaneous coronary intervention, and 10 patients had received coronary artery bypass graft surgery.

**Table 1:** Characteristics of study participants.

Characteristics	
Continuous variables	Mean (SD)
Age (years)	53.53 (12.47)
BMI (kg/m^2^)	26.54 (4.41)
Categorical variables	*N* (%)
Body mass index (kg/m^2^)	
<18.5	3 (3.8%)
18.5∼23.9	16 (20.25%)
24.0∼27.9	40 (50.63%)
≥28.0	20 (25.31%)
Coronary heart disease	65 (82.27%)
Myocardial infarction	36 (45.56%)
Arrhythmias	17 (21.51%)
Heart valve disease	5 (6.32%)
Hypertension	39 (49.37%)
Diabetes	12 (15.19%)
Percutaneous coronary intervention	32 (40.51%)
Coronary artery bypass graft	10 (12.66%)

### 3.2 Physical performance

Table 2 displays the results of our study. After twelve sessions of CR, there was a significant increase in VO_2_ at AT, (10.9 ± 2.9 to 13.3 ± 3.4 mL/min/kg, *p* < 0.01). Notably, heart rate remained relatively stable and did not show a marked change at the AT. Additionally, VE at AT showed a significant difference (29.3 ± 7.5 to 35.6 ± 9.3 mL/min/kg, *p* < 0.01). The remarkable increase in bicycle power at AT was also observed (56.7 ± 25.4 to 70.0 ± 27.7, *p* < 0.01).

**Table 2:** CPET parameters at pre- and post-rehabilitation in different groups.

	Overall					BMI < 26.5 (*n* = 39)					BMI > 26.5 (*n* = 40)				
	pre-CR		post-CR		pre- vs. post-CR *p* (*t* Test)	pre-CR		post-CR		pre- vs. post-CR *p* (*t* Test)	pre-CR		post-CR		pre- vs. post-CR *p* (*t* Test)
	Mean	SD	Mean	SD		Mean	SD	Mean	SD		Mean	SD	Mean	SD	
Anaerobic threshold (AT)															
HR (cpm)	104.9	15.8	106.2	14.0	0.32	107.1	3.1	107.5	2.4	0.84	102.6	10.3	105.0	13.4	0.21
VO_2_ (mL/min/kg)	10.9	2.9	13.3	3.4	<0.01*	11.1	2.9	13.1	3.4	<0.01*	10.7	2.9	13.4	3.5	<0.01*
VE (L/min)	29.3	7.5	35.6	9.3	<0.01*	26.7	6.9	33.2	8.9	<0.01*	31.8	7.2	38.9	9.2	<0.01*
Load (W)	56.7	25.4	70.0	27.7	<0.01*	48.2	23.9	61.2	26.1	<0.01*	64.9	24.5	78.6	26.8	<0.01*
Slope															
VE/VCO_2_	30.7	5.2	28.8	4.2	<0.01*	32.4	6.3	29.9	5.2	<0.01*	29.1	3.3	27.7	2.51	<0.01*
OUES	1,372.4	340.0	1,553.5	402.9	<0.01*	1,222.6	332.0	1,384.1	399.9	<0.01*	1,514.7	284.7	1,728.7	324.3	<0.01*

* *p* < 0.05.

SD, standard deviation; cpm, counts per minutes; HR, heart rates; VO_2_, oxygen uptake; VE, ventilation; OUES, oxygen uptake efficiency slope.

**Figure 2:**
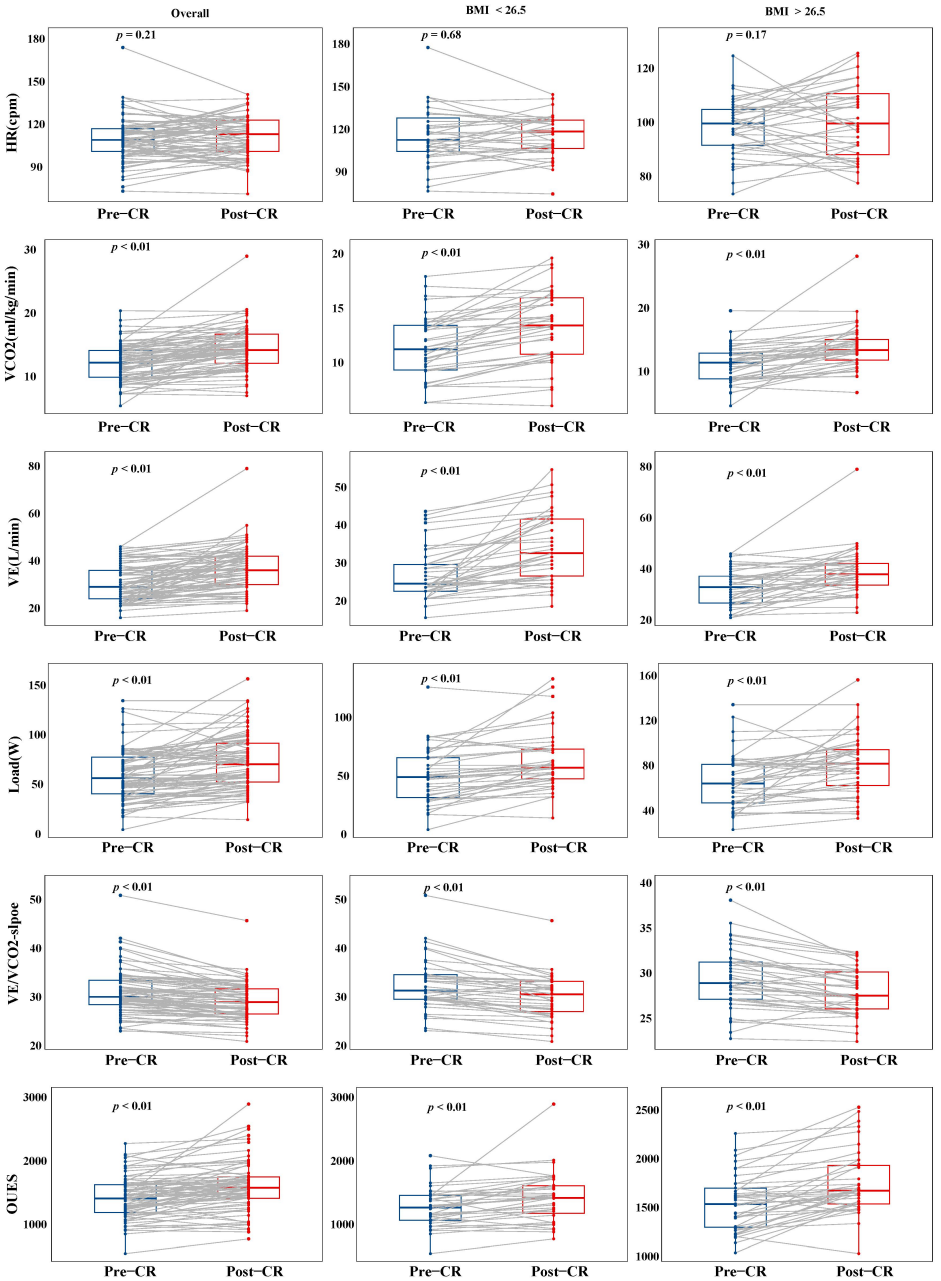
Distribution of significantly different variables in CPET between pre-CR and post-CR in all participants, the BMI < 26.5 group and the BMI > 26.5 group. HR, heart rate; VO_2_, oxygen uptake; VE, ventilation; OUES, oxygen uptake efficiency slope; AT, anaerobic threshold.

To investigate the impact of BMI on CR response and to maximize statistical power given our sample size, participants were divided into two equal-sized groups using the median BMI (26.5 kg/m^2^). This approach allows for a balanced comparison, as using standard WHO BMI categories (e.g., normal, overweight, obese) would have resulted in highly unequal group sizes (*n* = 19, 40, 20, respectively). The low-BMI group consisted of 39 patients (25 male), while the high-BMI group comprised 40 patients (37 male). Significant increases in VO_2_ at AT were observed in both groups (*p* < 0.01). Further details regarding the significant differences in CPET results are presented in Figure 2, which includes parameters such as VE at AT, VO_2_ at AT, VE/VCO_2_-slope, and OUES.

### 3.3 Energy expenditure and quality of life

**Figure 3:**
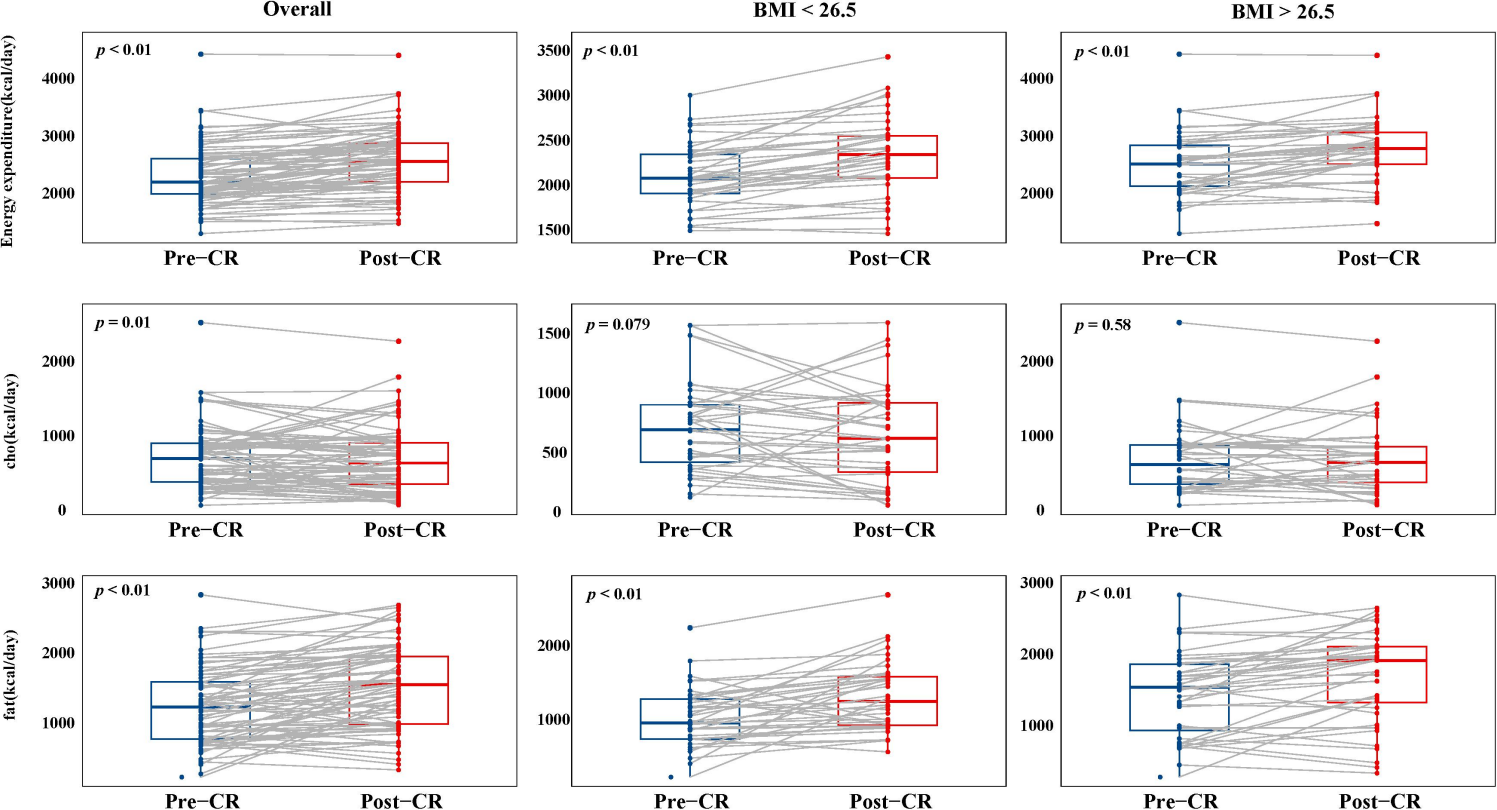
Total energy expenditure, carbohydrate energy, and fat energy. cho, carbohydrate energy; fat, fat energy.

**Figure 4:**
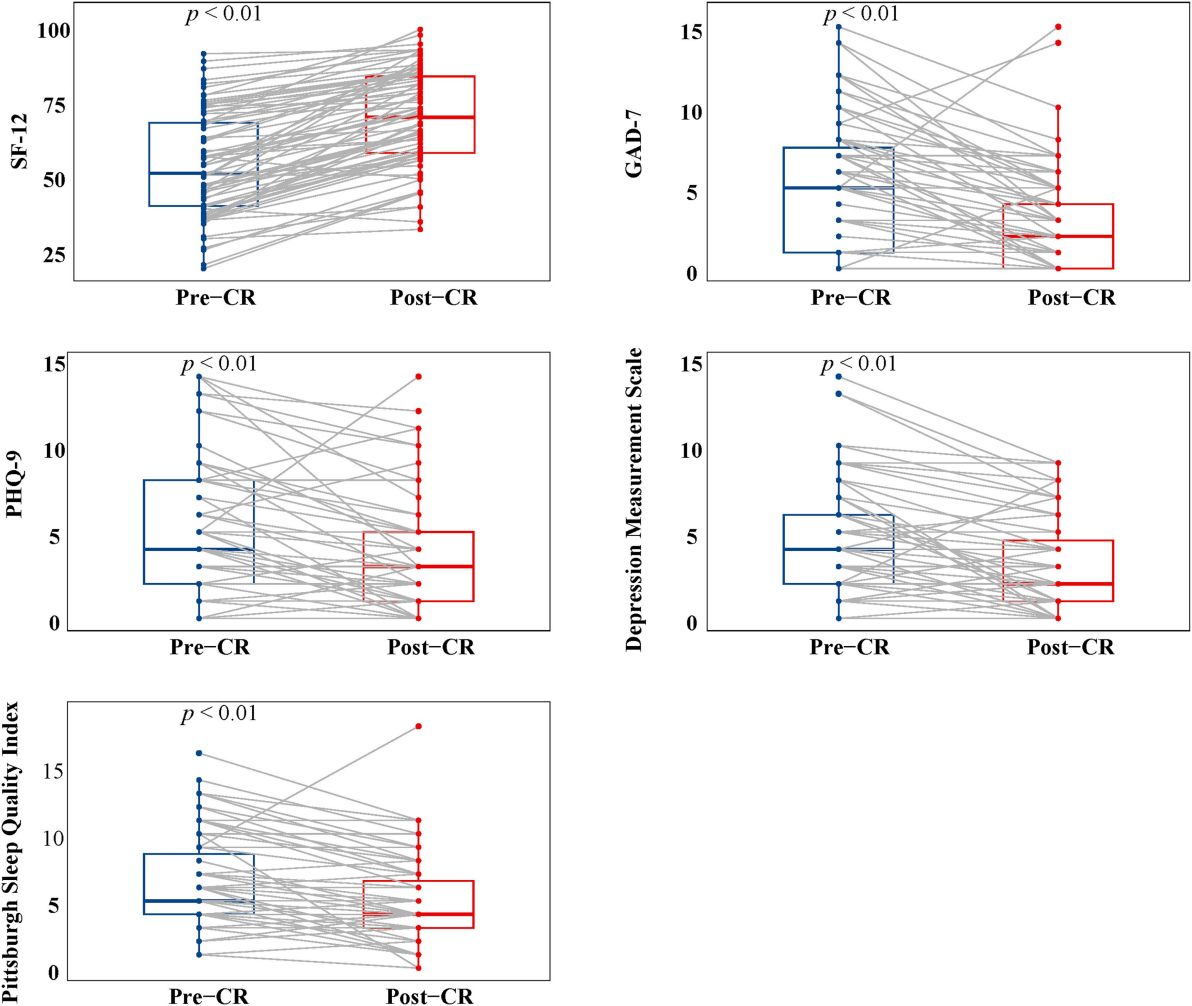
Comparison of quality of life, depression and anxiety and sleep quality. SF-12,12-item Short Form Survey; GAD-7, Generalized Anxiety Disorder 7-item; PHQ-9, Patient Health Questionnaire-9.

After 12 sessions of CR, there was a significant increase in EE, and this change was mainly due to a significant increase in fat consumption instead of cho in different groups (Figure 3). Moreover, as shown in Figure 4, the score of SF-12 significantly increased after CR (*p* < 0.01). The level distribution of GAD-7 and PHQ-9 also varied significantly (*p* < 0.01). Depression scores and sleep quality index were also significantly decreased as measured by the corresponding scales (*p* < 0.01).

## 4 Discussion

Our study demonstrates that a 12-session, exercise-based cardiac rehabilitation CR program significantly improves cardiorespiratory fitness, resting energy expenditure, quality of life, and psychological well-being in patients with CVD. Importantly, these benefits were observed across both low—and high—BMI subgroups.

### 4.1 Improvements in cardiorespiratory fitness

We observed a 21.2% increase in oxygen uptake (VO₂) at the anaerobic threshold (AT), along with significant rises in workload and ventilation at AT. Unlike peak VO₂, which requires maximal exertion, VO₂ at AT is safer and more feasible in CVD patients (23, 24). This submaximal improvement is clinically meaningful because even moderate gains in exercise capacity are linked to better long-term prognosis (22).

Recent trials confirm the importance of exercise intensity: high—intensity training, even over relatively short durations, yields the most substantial improvements in cardiorespiratory fitness*,* and HIIT appears particularly effective, especially when combined with resistance training (25, 26). A 2025 randomized controlled trial in patients with chronic heart failure after TAVR reported that individualized exercise-based CR significantly improved AT, peak VO₂, and quality of life compared with conventional rehabilitation (27), reinforcing the strategy used in our study.

The VE/VCO₂ slope decreased significantly from 30.7 to 28.81. A 2022 study found that this metric is a strong prognostic indicator across the heart failure spectrum, and values below 30 are considered normal (25, 26). Our post-intervention value (28.81) thus reflects restored ventilatory efficiency.

OUES increased by 13.2% overall, with a larger gain in the high-BMI group (+14.1%). A 2024 study of 82 CHD patients reported that a 3-month aerobic training program induced a 5.0% cut-off for identifying high responders, with peripheral and ventilatory adaptations (increased VE and arteriovenous oxygen difference) being key determinants of VO₂ peak improvement (28). Our OUES findings align with this observation.

### 4.2 Metabolic benefits

Resting energy expenditure (EE) increased significantly after CR, driven primarily by enhanced fat oxidation rather than carbohydrate utilization. A review on exercise intensity and cardiac metabolic adaptation noted that structured exercise improves metabolic efficiency while reducing sedentary behavior, with myokines such as irisin playing a role in improving fat oxidation and glucose metabolism (22)*.* An integrative physiology review further explained that exercise protects the heart by improving metabolism, reducing inflammation and cell damage, and strengthening connections between heart cells and blood vessels (29). These mechanisms likely underlie the metabolic shift observed in our study.

### 4.3 Quality of life and psychological outcomes

After CR, patients showed significant improvements in quality of life (SF-12) and reductions in anxiety (GAD-7) and depression (PHQ-9). These psychological benefits are well supported by existing evidence. A 2021 Cochrane review (30 trials, *n* = 4,873) found that exercise-based CR reduces depression in coronary heart disease patients (22), and an updated 2025 Cochrane review (24 trials, *n* = 5,091 with heart failure) confirmed reductions in depression and heart failure-related hospitalizations (30). Similarly, a 2024 meta-analysis by Gupta et al. (22 trials, *n* = 2,237) reported significant improvements in both physical and mental components of health-related quality of life following exercise-based CR (31). Collectively, these findings indicate that CR confers meaningful psychological benefits that complement its physical advantages in improving long-term outcomes.

### 4.4 Effects across BMI categories

A key finding is that both low-BMI (<26.5) and high-BMI (>26.5) groups experienced comparable improvements in VO₂ at AT, VE/VCO₂ slope, OUES, EE, and QoL. However, recent evidence on the influence of BMI is nuanced. A study of 1313 women found that a 12-week CR program increased CRF in women with normal and overweight BMI, but those with obesity (BMI ≥ 30) did not realize similar improvements (32). Another study found that obese patients had higher baseline BMI and were more likely to have stable angina and OSA, but still showed improvements in CRF (27). Collectively, these recent findings indicate that while BMI influences CR outcomes, the effect is modifiable by sex, obesity severity, and body composition. Our study adds that for overweight and moderately obese patients (BMI up to ∼30), standard CR yields substantial benefits.

### 4.5 Clinical implications and guidelines

The improvements observed in our study align with recent guideline updates. The 2024 AHA/AACVPR scientific statement on core components of cardiac rehabilitation, published in *Circulation*, expanded the framework to include program quality, remote delivery models, and enhanced weight and psychosocial management (33). Similarly, the ESC's 2025 standards for cardiac telerehabilitation emphasize individualized, multidimensional rehabilitation strategies addressing physical, metabolic, and psychological health (34). Our findings support these recommendations by demonstrating that structured CR improves cardiorespiratory fitness, fat oxidation, and mental well-being across BMI categories, reinforcing that modern CR should be offered to all eligible CVD patients.

### 4.6 Limitations

This study has several limitations. First, the lack of a non-intervention control group limits causal inference, though withholding CR from eligible patients would raise ethical concerns. First, the lack of a non-intervention control group limits causal inference, though withholding CR from eligible patients would raise ethical concerns. Given the early stage of CR implementation in China and the ethical constraints, a pre-post design was a pragmatic alternative commonly accepted in feasibility studies (27, 30). Second, the intervention duration (12 sessions) and follow-up (only post-intervention) preclude assessment of long-term sustainability. Although international guidelines typically recommend 24–36 sessions, the 12-session regimen used here was chosen based on the early stage of CR implementation in China, where shorter protocols are clinically feasible and have been shown to yield meaningful short-term benefits (35, 36). Third, potential selection bias exists given the single-center design and male-predominant sample (78.5%). Fourth, we did not report adjustments to cardiovascular medications during the study, which may influence physiological outcomes. Notably, several of these limitations reflect the early stage of cardiac rehabilitation in China, where structured outpatient CR programs have only recently emerged and long-term follow-up infrastructure remains underdeveloped (35, 36). The focus on short-term feasibility and safety is therefore a necessary first step in this context. Future multicenter trials with extended follow-up and medication monitoring are warranted.

## 5 Conclusion

In summary, a short-term exercise-based CR program substantially improves cardiorespiratory fitness (VO₂ at AT, OUES, VE/VCO₂ slope), shifts resting metabolism toward fat oxidation, enhances quality of life, and reduces anxiety and depression in CVD patients. These benefits are independent of BMI, supporting the broad implementation of CR across the entire weight spectrum.

## Data Availability

The raw data supporting the conclusions of this article will be made available by the authors, without undue reservation.
